# A Camera Intrinsic Matrix-Free Calibration Method for Laser Triangulation Sensor

**DOI:** 10.3390/s21020559

**Published:** 2021-01-14

**Authors:** Xuzhan Chen, Youping Chen, Bing Chen, Zhuo He, Yunxiu Ma, Dailin Zhang, Homayoun Najjaran

**Affiliations:** 1 School of Mechanical Science and Engineering, Huazhong University of Science and Technology, Wuhan 430074, China; chenxuzhan@hust.edu.cn (X.C.); ypchen@hust.edu.cn (Y.C.); hezhuo_mse@hust.edu.cn (Z.H.); mnizhang@hust.edu.cn (D.Z.); 2 School of Material Science and Engineering, Huazhong University of Science and Technology, Wuhan 430074, China; yunxiu_ma@hust.edu.cn; 3 School of Engineering, The University of British Columbia, Kelowna, BC V1V 1V7, Canada; homayoun.najjaran@ubc.ca

**Keywords:** calibration, laser triangular sensor, one-dimensional target

## Abstract

Laser triangulation sensors (LTS) are widely used to acquire depth information in industrial applications. However, the parameters of the components, e.g., the camera, of the off-the-shelf LTS are typically unknown. This makes it difficult to recalibrate the degenerated LTS devices during regular maintenance operations. In this paper, a novel one-dimensional target-based camera intrinsic matrix-free LTS calibration method is proposed. In contrast to conventional methods that calibrate the LTS based on the precise camera intrinsic matrix, we formulate the LTS calibration as an optimization problem taking all parameters of the LTS into account, simultaneously. In this way, many pairs of the camera intrinsic matrix and the equation of the laser plane can be solved and different pairs of parameters are equivalent for displacement measurement. A closed-form solution of the position of the one-dimensional target is proposed to make the parameters of the LTS optimizable. The results of simulations and experiments show that the proposed method can calibrate the LTS without knowing the camera intrinsic matrix. In addition, the proposed approach significantly improves the displacement measurement precision of the LTS after calibration. In conclusion, the proposed method proved that the precise camera intrinsic matrix is not the necessary condition for LTS displacement measurement.

## 1. Introduction

The laser triangulation sensor (LTS) is widely used to acquire depth information in industrial applications such as rail inspection [1], reverse engineering [2], and workpiece inspection [3]. In industrial environments, collisions and impacts can often change the parameters of the LTS setups, compromising the performance and measurements of the sensors. Thus, LTS calibration is an important problem for increasing the measurement precision.

The LTS calibration methods can be categorized as pattern-based methods, target geometry-based methods, and auxiliary device-facilitated methods according to their different calibration principles. Thanks to the well-developed machine vision methods for corner and line detection, the use of a carefully designed pattern is an effective approach for localizing the 2D planar target. The chessboard pattern is widely used to determine the extrinsic parameters of the camera [4], and then the laser plane can be fitted using optimization [2,5,6]. Since the accuracy of the corner detection limits the localization precision for the chessboard pattern, the camera coordinate and pixel coordinate are optimized together to solve the problem [7]. Besides, a front coated mirror is used to improve the localization precision of the chessboard target [8]. Recently, various patterns such as parallels [9,10] and circles [11,12] are proposed to improve the target localization precision. Although researchers have made great progress on pattern-based LTS calibration, there exist two main drawbacks to the pattern-based methods. First, in order for the camera to capture the patterns on the targets, the targets must have relatively large physical sizes. Second, the LTS systems used in the industry have optical filters to omit the ambient light. This can make the patterns unrecognizable under nonideal lighting conditions which is likely in industrial environments.

The industry demands on-site LTS calibration methods that can operate in narrow spaces and in limited time [1]. To solve the on-site LTS calibration problem, the target geometry-based methods are developed that use compact targets such as cylinders [13], ball [14], and 1D colinear balls [15], and multitooth gauge [1]. However, the compact target-based methods [1,13,14,15] assume that the exact camera intrinsic matrix is available. Thus, a slight deviation of the camera intrinsic matrix will significantly impact the measurement precision.

Although some camera intrinsic matrix-free methods are proposed [16,17,18,19,20], the auxiliary devices such as 3D chessboard boxes [16,17,18,19] and guide rails [20] that have to be fixed on the ground are used in the calibration process. The need for such auxiliary devices makes the camera intrinsic matrix-free methods [16,17,18,19,20] impractical for on-site calibration.

In this paper, a novel camera intrinsic matrix-free method is introduced that omits the need for auxiliary devices. The proposed method uses a compact one-dimensional (1D) target that can move freely on the laser plane to solve the LTS calibration problem. The designed 1D target has three colinear feature points and the LTS can be calibrated based on the geometry constraint of the target. In contrast to the traditional methods that rely on the precise camera intrinsic matrix [1,2,4,5,6,7,8,9,10,11,12,13,14,15] or methods that use auxiliary devices [16,17,18,19,20], we formulate the LTS calibration as an optimization problem without the need for either of them. More precisely, many equivalent pairs of the camera intrinsic matrix and the equation of the laser plane can be solved by optimization, simultaneously. Thus, the proposed method can calibrate the LTS without knowing the intrinsic matrix of the camera so it is suitable for off-the-shelf LTS used in industrial applications. The results show that the proposed approach significantly improves displacement measurement precision of the LTS after calibration.

The novelties of the proposed method are as follows.

(i) A new model that simultaneously optimizes the camera intrinsic matrix and the equation of the laser plane, instead of calculating the equation of the laser plane based on the known camera intrinsic matrix.
(ii) A novel accurate and computationally efficient closed-form solution of the position of the colinear 1D target.

After presenting the proposed method in the following section, the results of several simulation and real-world experiments will be presented to verify the efficacy of the proposed method.

## 2. Related Works

### 2.1. Calibrating the LTS Based on the Patterns of the Targets

The patterns on the calibration targets are used to localize the targets in the camera coordinate system. The chessboard patterns are widely used to localize the calibration target in the camera coordinate system [4,7,8]. In this way, the calibration target can be localized using corner detection results. However, the corner detection error of the chessboard target affects the LTS calibration. Some researchers have improved the chessboard pattern target-based LTS calibration methods by optimizing the laser plane fitting strategies. For example, Xu et al. [5] used the Plauck matrix to optimize the solution of the laser plane. Hence, the laser plane fitting is less affected by the noise amplitude and number of images. Xu et al. [2], the authors estimated the laser plane using maximum likelihood estimation, and their experimental results show that the relative error is reduced by about 20%. Xu et al. [6], the laser plane is optimized using the RANSAC algorithm. In this way, the error of laser stripe extraction is countered and the LTS calibration is improved.

In addition to optimizing the plane fitting strategy, some researchers have worked on improving target pattern recognition. Wei et al. [9] designed the parallel pattern for the 2D planar target. The orientation of the target can be calculated based on the vanishing point i.e., the orientation of the target is calculated more precisely. Continuing with [9], Wei et al. [10] then improved the precision of the distance from the camera to the laser plane for the parallel pattern planar target. Motivated by the fact that the parallel line pattern introduces projection errors, Shao et al. [11] designed a concentric circle pattern that can reduce the perspective deviation and improved the LTS measurement precision.

### 2.2. Calibrating the LTS Based on Target Geometries

Compared to the pattern-based LTS calibration methods that require a relatively large plane, compact targets with various geometries are drawing more attention from the researchers. Wei et al. [15] proposed a 1D target-based method that calibrates the LTS by detecting three colinear feature points from the 1D target. Although the 1D target is compact and suitable for on-site calibration, solving for the target poses based on a numerical solution is computationally expensive. Liu et al. [13] proposed a single ball target-based calibration method. It is suitable for on-site narrow space LTS calibration since the proposed method is flexible and the single ball target is compact [14]. Building on their previous work, Liu et al. [13] proposed a parallel cylinder-based LTS calibration method that calculates the equation of the laser plane based on the minor axis of an ellipse. In contrast to Liu et al. [14], Liu et al. [13] only requires the image of the laser stripe to calibrate the system and is suitable for the LTS with the optical filter.

Recently, Zhu et al. [21] calibrated the LTS using a cylinder target. The advantage of Zhu et al. [21] is that there is no special requirement for the position of the cylinder target. Wu et al. [12] designed a slot-like calibration target, from which the fiducial points can be easily extracted. With the characterized geometry, the minimum geometric accuracy is satisfactory (18 um). Pan et al. [1] proposed a multitooth target-based method that is independent of feature points and unaffected by external light. Thus, Pan et al. [1] is suitable for on-site calibration.

### 2.3. Calibrating the LTS with Auxiliary Devices

Auxiliary devices can facilitate LTS calibration methods. For example, they omit the need for recognizing the patterns in the camera images. Huang et al. [20] calibrated the LTS using an on-rail combined gauge. The combined gauge has a relatively small size, but it has to be fixed on a guide rail. Thus, it lacks flexibility for on-site calibration. A series of LTS calibration methods that use geometry targets are proposed by Xu’s group [16,17,18,19]. A common feature of [16,17,18,19] is that a 3D chessboard box is used to solve the extrinsic parameters of the camera. In this way, the laser plane can be localized by intersecting with height gauges [16,17] and end-fixed sticks [18,19]. The drawback of [16,17,18,19] is that the 3D chessboard box has a relatively large size.

## 3. Method

**Figure 1:**
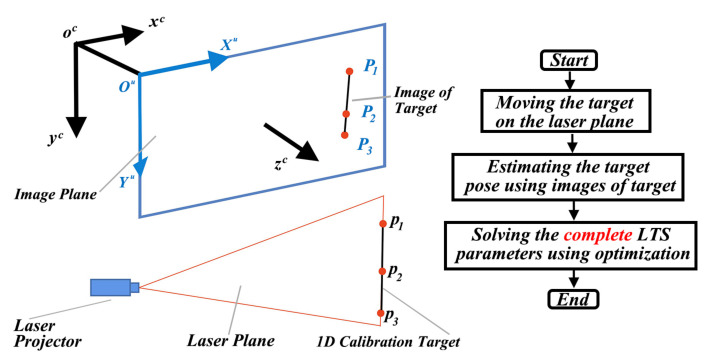
The schematic of the proposed laser triangulation sensors (LTS) calibration method.

The proposed method uses a one-dimensional (1D) target with three colinear feature points to calibrate the LTS. During calibration, we first move the target on the laser plane and capture images. Then, the feature points are detected from the images. The positions of the target are estimated based on the proposed closed-form solution. The coplanar constraint of the target is used to solve the parameters of LTS. The schematic diagram of the proposed LTS calibration method is shown in Figure 1.

### 3.1. The Closed-Form Solution for Localizing the 1D Target

In Figure 1, the camera coordinate system is denoted as $o^{c}x^{c}y^{c}$ and the image coordinate system is denoted as $O^{u}X^{u}Y^{u}$. The feature points of the target are denoted as $p_{1},p_{2},p_{3}$. The corresponding projected points on the image are denoted as $P_{1},P_{2},P_{3}$.

**Figure 2:**
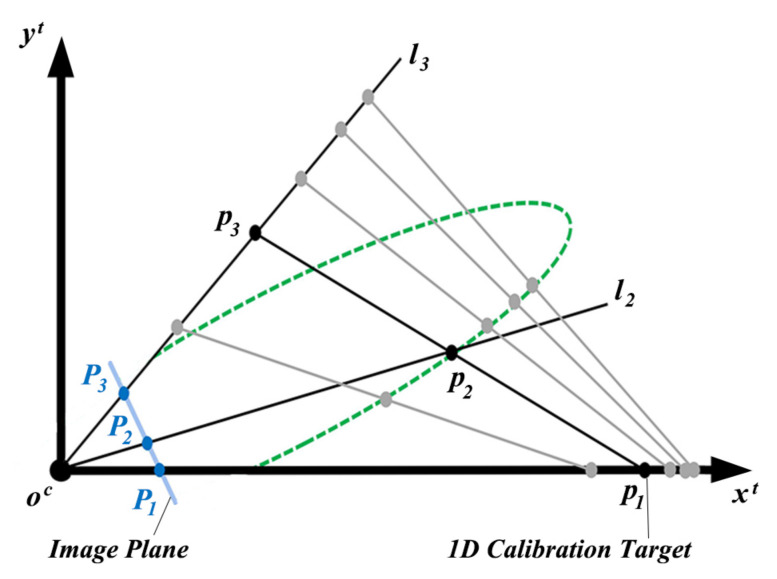
2D local coordinate system on the 2D plane spanned by the camera optical center and the 1D target.

To calculate the position of the target, a 2D local coordinate system $o^{c}x^{t}y^{t}$ is established on the 2D plane that is spanned by the camera optical center $o^{c}$ and the 1D target shown in Figure 2. In Figure 2, the line connecting $o^{c}$ and $p_{1}$ is set as the *x* axis of $o^{c}x^{t}y^{t}$. The line connecting $o^{c}$ and $p_{2}\left(x_{2},y_{2}\right)$ is denoted as $l_{2}$. The line connecting $o^{c}$ and $p_{3}\left(x_{3},y_{3}\right)$ is denoted as $l_{3}$. The slopes of $l_{2}$ and $l_{3}$ are denoted as $k_{2}$ and $k_{3}$, respectively.

The length of the calibration target (*L*) is used to restrain the position of the target,

(1) $${\left(x_{1}-x_{3}\right)}^{2}+{y_{3}}^{2}=L^{2}$$

Figure 2 marks four different target positions that satisfy Equation (1). These target positions are shown in grey. λ is the ratio of the distance between $p_{1}$ and $p_{2}$ to the length of the target and can be calculated as,

(2) $$\lambda =\frac{\left|\right|p_{1}p_{2}\left|\right|}{L}$$

where $\left|\right|p_{1}p_{2}\left|\right|$ is the distance between target feature point $p_{1}$ and $p_{2}$.

Then $x_{1}-x_{3}$ and $y_{3}$ can be calculated as follows.

(3) $$x_{1}-x_{3}=\frac{x_{2}}{\lambda }-\frac{y_{2}}{k_{3}\left(1-\lambda \right)\lambda }$$

(4) $$y_{3}=\frac{y_{2}}{1-\lambda }$$

Combining Equations (1), (3) and (4), we find that $p_{2}$ that satisfies Equation (1) is on an elliptical curve that is marked by the green dash line in Figure 2. $p_{2}$ is in the intersection of $l_{2}$ and the elliptical curve. $l_{2}$ can be calculated as,

(5) $$y_{2}=k_{2}\cdot x_{2}$$

Then position of $p_{2}$ in $o^{c}x^{t}y^{t}$ can be calculated as,

(6) $$p_{2}=\left[\begin{array}{c} x_{2}\\ y_{2} \end{array}\right]=\left[\begin{array}{c} \frac{L}{\sqrt{{\left(\frac{k_{2}}{1-\lambda }\right)}^{2}+{\left(\frac{k_{3}\left(1-\lambda \right)-k_{2}}{k_{3}\left(1-\lambda \right)\lambda }\right)}^{2}}}\\ k_{2}x_{2} \end{array}\right]$$

Based on the position of $p_{2}$ and λ, we can solve the position of $p_{1},p_{3}$ in $o^{c}x^{t}y^{t}$,

(7) $$p_{3}=\left[\begin{array}{c} x_{3}\\ y_{3} \end{array}\right]=\left[\begin{array}{c} \frac{y_{2}}{k_{2}\left(1-\lambda \right)}\\ \frac{y_{2}}{1-\lambda } \end{array}\right]$$

(8) $$p_{1}=\left[\begin{array}{c} x_{1}\\ 0 \end{array}\right]=\left[\begin{array}{c} x_{3}+\frac{x_{2}-x_{3}}{\lambda }\\ 0 \end{array}\right]$$

$k_{2}$ and $k_{3}$ can be calculated based on the angles between $l_{2}$, $l_{3}$ and the *x* axis, respectively,

(9) $$k_{i}=\tan\cos^{-1}\left(\frac{A^{-1}P_{i}\cdot A^{-1}P_{1}}{∥A^{-1}P_{i}∥∥A^{-1}P_{1}∥}\right)$$

where i=2,3 and *A* is the intrinsic matrix of the camera that can be represented as,

(10) $$A=\left[\begin{array}{ccc} f_{x} & 0 & u\\ 0 & f_{y} & v\\ 0 & 0 & 1 \end{array}\right]$$

where $f_{x},f_{y},u,v$ are internal parameters of the camera intrinsic matrix. Those parameters can be obtained by calibration or calculated based on the camera specifications.

Since the distances from $p_{1},p_{2}$ and $p_{3}$ to $o^{c}$ are equivalent in $o^{c}x^{c}y^{c}$ and $o^{c}x^{t}y^{t}$, the formulations of $p_{1},p_{2}$ and $p_{3}$ in $o^{c}x^{c}y^{c}$, denoted as $p_{1}^{c},p_{2}^{c}$ and $p_{3}^{c}$ can be calculated as,

(11) $$p_{1}^{c}=\frac{∥o^{c}p_{1}∥}{∥A^{-1}P_{1}∥}A^{-1}P_{1}$$

(12) $$p_{3}^{c}=\frac{∥o^{c}p_{3}∥}{∥A^{-1}P_{3}∥}A^{-1}P_{3}$$

(13) $$p_{2}^{c}=p_{1}+\lambda \left(p_{3}^{c}-p_{1}^{c}\right)$$

where ||·|| denotes distance.

In this way, we can solve the position of the target in a closed-form fashion and the information required includes the camera intrinsic matrix *A*, the target length *L*, and the ratio λ.

### 3.2. Solving the LTS Parameters Based on the Optimization

The equation of the laser plane is given by,

(14) a+bx+cy+z=0

where a,b,c are the parameters of the plane and the points p(x,y,z) are the feature points of the target which are on the plane if they satisfy Equation (14).

The fitting error of plane is used to calculate the cost function of optimization where the error can be calculated as,

(15) $$e={\left[p\cdot {\left[a,b,1,c\right]}^{T}\right]}^{T}\cdot \left[p\cdot {\left[a,b,1,c\right]}^{T}\right]$$

where $p={\left[p_{1},p_{2},p_{3},\ldots ,p_{i},\ldots ,p_{n}\right]}^{T}$ is the array of the homogeneous coordinates of the feature points. *e* is the error of plane fitting. When *e* is minimized to 0, *p* contains an array of coplanar points. In this way, the camera intrinsic matrix and the equation of the laser plane can be solved by,

(16) A,a,b,c=argmin(e)

Equation (16) can be solved using well-developed optimization methods such as the Trust Region method, which is the method used in our paper.

In contrast to a conventional LTS calibration method that solves the camera intrinsic matrix and the equation of the laser plane separately, we optimize on the camera intrinsic matrix and the equation of the laser plane simultaneously. However, the solution of the point array **p** is not unique since Equation (15) is satisfied when **p** is translated or rotated on the laser plane. Thus, Equation (16) is nonconvex and has several extreme points. In this way, the proposed method can find several pairs of camera intrinsic matrix (*A*) and the equation of the laser plane (a,b,c) based on the different initial values for solving the cost function. The experiments confirmed that the pairs of parameters are equivalent to LTS.

## 4. Experiments

### 4.1. Simulation of the Proposed LTS Calibration Method

Simulations are conducted to analyze the influence of the target length and the number of images on the proposed method. In the simulation, we used a pin-hole model to simulate the camera, the width of the output image is 1600 pixels and the height is 1200 pixels. The camera has 5 um pixels. The focal length of the camera 6 mm and the optical axis passes through the geometrical center of the image plane. The equation of the laser plane in the camera coordinate frame is −y+z−400=0.

**Figure 3:**
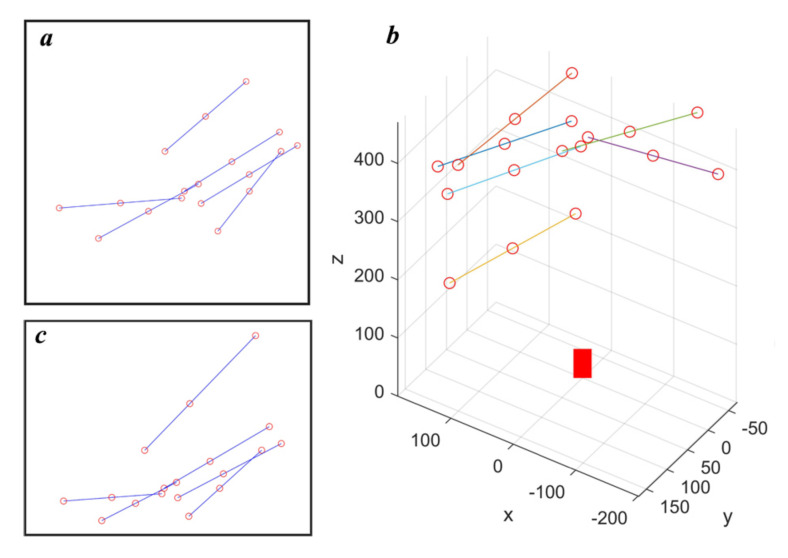
Generating data for calibration and verification in the simulation environment. (**a**) is the targets generated on the simulated laser plane. (**b**) shows the distribution of the targets in the 3D space. (**c**) is the image of the targets.

The calibration target has 3 feature points and the second feature point divides the target from the middle. In the simulation, the target positions are generated by randomly selecting one point on the plane, and randomly selecting the angle of target orientation, shown in Figure 3a. Then, the plane is transformed to the camera coordinate system based on the equation of the laser plane, shown in Figure 3b where the red block is used to represent the camera. The feature points are projected onto the image based on the pin-hole model of the camera. Figure 3c visualized the feature point projection results. The error of feature point detection is simulated by adding Gaussian noise (variance equals 0.5) to projection results. The source code of simulation can be found at https://github.com/RustIron/caminmatfreeLTSCalib.git.

We uniformly sampled 2000 pairs of points on the laser plane as the test dataset to evaluate the measurement precision of the calibrated LTS. The distances between the pair of points range from 10 mm to 400 mm, and the distances are recorded as the ground-truth value of the test dataset.

**Figure 4:**
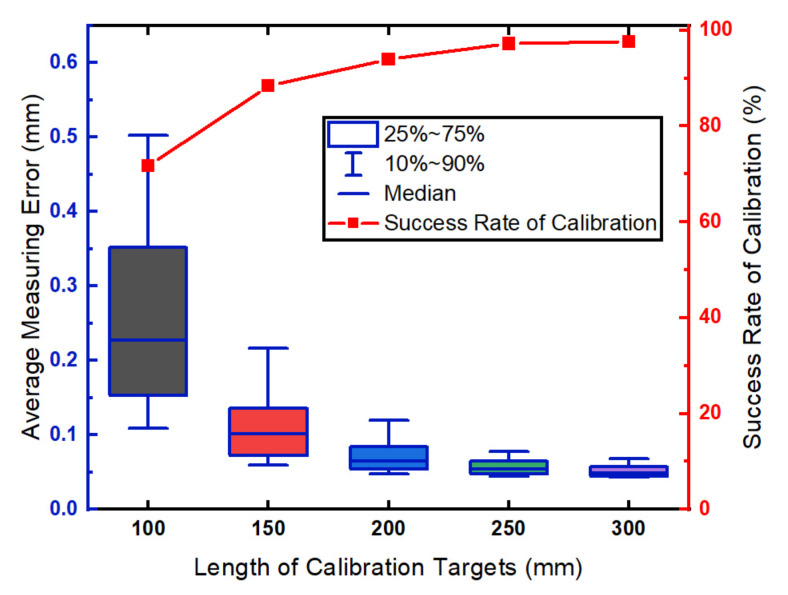
The influence of the length of calibration targets.

Five targets with different lengths (100 mm, 150 mm, 200 mm, 250 mm, 300 mm) are used to analyze the influence of the target lengths. For each length of the target, 12 images are used to calibrate the simulated LTS. The calibration and evaluation procedures are repeated 500 times. The LTS is successfully calibrated when the optimization function can find a minimum value that is less than 1. The relationship between the measurement precision of the LTS and the target lengths is shown in Figure 4.

Shown in Figure 4, the average measurement error is less than 0.1 mm when the target length is greater than 200 mm. In the configuration of the simulation, the Gaussian noise with a fixed variance is added to the projection of the feature points. According to Equation (6), the Gaussian noise affects the estimated $k_{1}$ and $k_{2}$ and the distance between the projected points $P{}_{i}P{}_{1}$ increases with the target length *L*. In this way, the longer the target the relatively less the influence on $k_{1}$ and $k_{2}$. Thus, the measurement errors decrease as the length of the calibration targets increase. The success rate of calibration significantly increases with the length of the calibration target. We attribute the increasing success rate of calibration to higher precision in the estimation of the target positions. The error of the target localization makes it hard, or even impossible, to find the parameters of the LTS using optimization.

**Figure 5:**
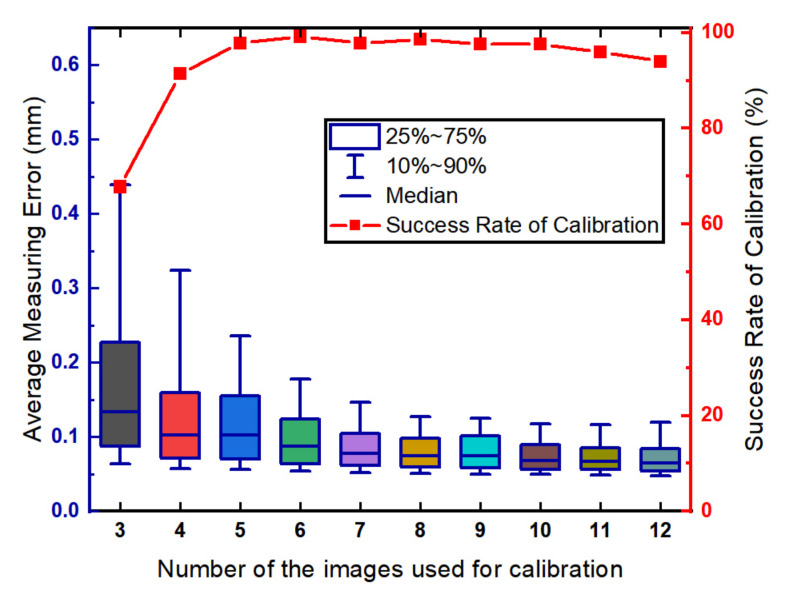
The influence of the number of the images.

We analyzed the influence of number of the images used for calibration. In the simulation, the number of images uses in the simulation ranged from 3 to 12. The length of the calibration target is 200 mm. The calibration and evaluation procedures are repeated 500 times for each round of the simulation. The relationship between the measurement precision of the LTS and the number of images is shown in Figure 5.

Shown in Figure 5, the average measurement error is less than 0.1 mm when the number of the images is greater than 6. The experimental results show that the measurement error decreases with the increase of the number of the images. Shown in Equation (16), the optimization goal is set as the sum of the square of the error. Increasing the number of the images will fit the plane with more points resulting in a better plane estimation. However, the success rate of calibration begins to decline slowly when the number of images is greater than 8. The reason is that increasing the number of the target poses can increase the risk of incorrect detection of the feature points. The relatively large error of feature point detection results in the incorrect estimation of the points on the plane, i.e., outliers. In this way, the plane estimation results can be unsatisfactory.

### 4.2. Real-World Experiment on the Proposed LTS Calibration Method

**Figure 6:**
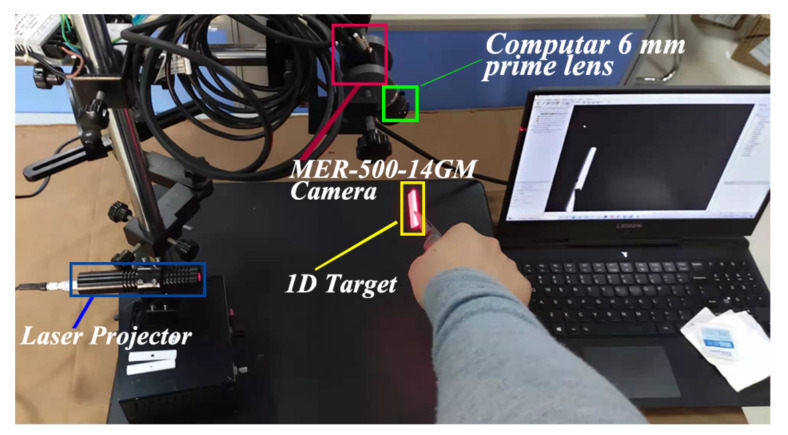
The apparatus used for the experiments.

We conducted real-world experiments to verify the proposed LTS calibration method. The experimental apparatus is shown in Figure 6. The type of the camera is MER-500-14GM that has 2.2 um pixels and the size of the output image is 2592×1944. A 6 mm prime lens is mounted on the camera. The wavelength of the laser projector is 650 nm.

**Figure 7:**
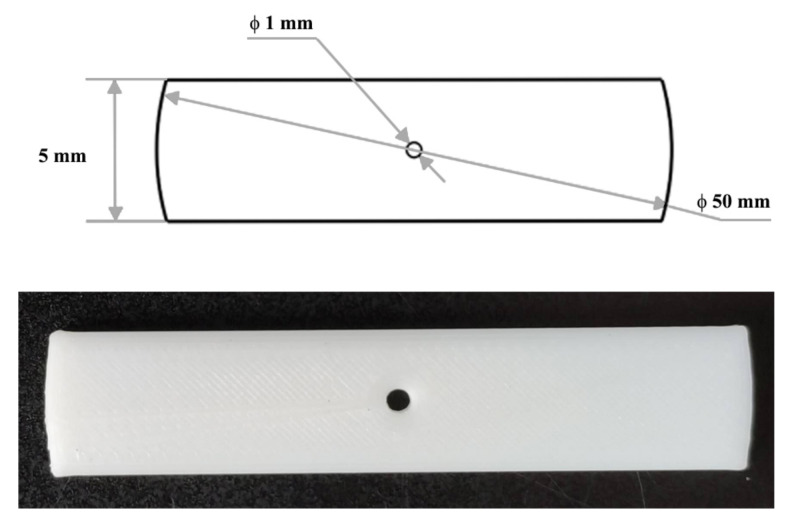
The calibration target used in the experiment.

The one-dimensional calibration target shown in Figure 7 is manufactured with a high precision 3D printing machine. Both ends of the target are arced with a radius of 25 mm, and the center of the arc is the small hole in the middle of the target. In this way, the three colinear feature points (two ends and the middle hole) can be detected if the laser stripe passes through the hole in the middle, i.e., the distances between the feature points are invariant even if the target has a small tilt during the calibration.

**Figure 8:**
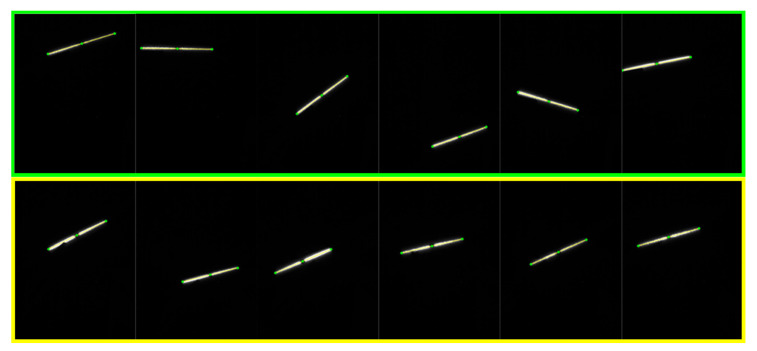
The images used for the LTS calibration and test.

We collected images to test the proposed LTS calibration method. Six images of the target (marked in green box) are used for the calibration and another six images (marked in yellow box) are used to verify the measurement precision of the apparatus. The laser stripes are detected using the Steger’s method [22]. In this way, the feature points on the two ends and middle of the target can be detected from the images, shown in Figure 8.

The calibrated parameters of the LTS are shown in Table 1. In the experiments, the LTS is calibrated using the proposed method and 4 pairs of parameters are generated using different initializing values of the camera (denoted as Parameter No. 1, Parameter No. 2, Parameter No. 3, and Parameter No. 4 in Table 1). To evaluate the precision of the proposed method, we calculated the camera intrinsic matrix and the equation of the laser plane based on the camera specification (denoted as Parameter Specification in Table 1).

The distances between the feature points of the targets are calculated to evaluate the proposed method. In the experiment, we compared the measurement results of different pairs of the parameters. In addition, the measurement precision of the calibrated parameters is compared with the counter part of the parameters calculated based on the camera specifications. The measurement results are shown in Table 2.

The measurement results of Parameter No. 1, Parameter No. 2, Parameter No. 3, and Parameter No. 4 have negligible differences (less than 0.01 mm). The measurement errors of the single measurement mainly come from the position error of the target, i.e., the center of the target is not exactly on the laser plane. However, the results of the experiment shows that different pairs of the LTS parameters are equivalent. It means that the precise camera intrinsic matrix is not the necessary condition for LTS displacement measurement.

**Figure 9:**
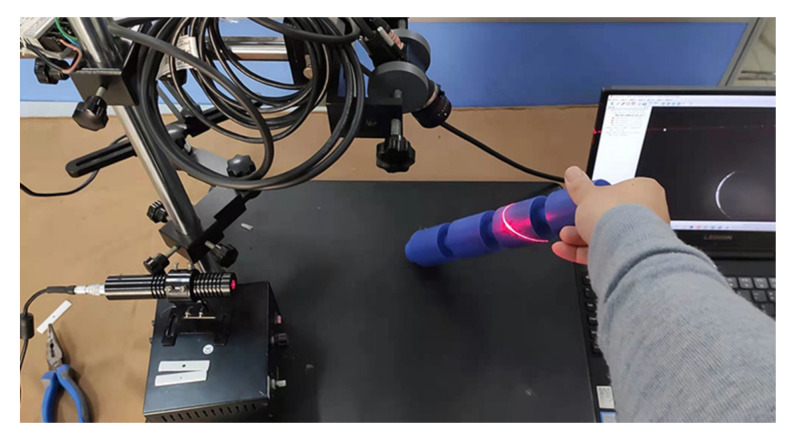
The cylinder measuring experiments.

In order to test the measurement precision of the proposed calibration method, a cylinder shown in Figure 9 is used as the measured object. The diameter of the cylinder is 45.0 mm. The laser plane intersects with cylinder and cross-section is an ellipse. Although the length of the ellipse along the minor axis is affected by the poses of the cylinder, the short axis length is invariant to the cylinder poses and equals to the diameter of the cylinder. In this way, the precision of the experiment apparatus can be evaluated.

**Figure 10:**
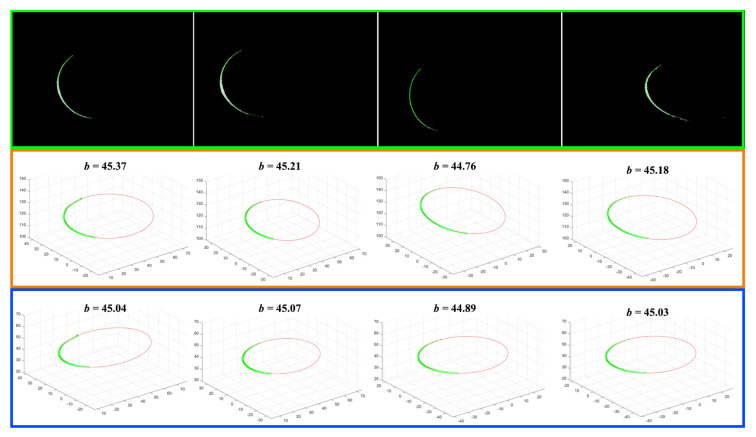
The results of cylinder measuring experiments.

The measurement results are shown in Figure 10. The laser stripes of the ellipse are detected with the Steger’s method Figure 10. Then, the laser stripes are reconstructed in 3D, and an ellipse is fitted using the least squares algorithm. The middle and bottom rows show the measurement results using Parameter Specification and Parameter No. 1, respectively where *b* is the estimated length of the short axis. In this way, the length of the short axis can be calculated.

The measurement error of the proposed method is less than 0.1 mm while the measurement error of LTS calibration using specification parameters is generally a few times larger. With the experimental results, it is reasonable to infer that the proposed method is suitable for improving the precision of the LTS without the need for the accurate camera intrinsic matrix. The measurement errors are resulted from two sources: (i) the precision of laser stripe detection (the surface reflection distorts the laser stripe since the cylinder surface was relatively shiny); and (ii) fitting errors of the ellipse (the cross-section is not a perfect ellipse since the laser stripe is distorted). The distortion error makes the least squares algorithm suboptimal.

## 5. Conclusions

In this paper, a novel one-dimensional target-based laser triangulation sensor (LTS) calibration method is proposed. An advantage of the proposed method is that it does not require the knowledge of the camera intrinsic matrix for calibration. The core contribution of this paper includes a new model of the LTS that simultaneously optimizes the camera intrinsic matrix and the equation of the laser plane and a closed-form solution for localizing the 1D target in the camera coordinate system. In this way, the LTS can be calibrated without the camera intrinsic matrix. Simulations show that the proposed LTS calibration method yields a better performance when a longer target and more images are used. Experiments on our apparatus show that the displacement measurement error is less than 0.1 mm and it is significantly reduced compared to calibrate the same apparatus with the device specifications. In summary, the proposed method can improve the measurement precision, simplify the calibration procedure, and proved that the precise camera intrinsic matrix is not the necessary condition for LTS displacement measurement. In the future work, we will focus on the extrinsic calibration of the LTS sensors that are mounted on a 6 DoF platform.

## Figures and Tables

**Table 1:** The calibration results.

Paramters	$f_{x}$	$f_{y}$	*u*	*v*	*a*	*b*	*c*
Parameter Specification	2727.27	2727.27	1296.00	972.00	125.622	−0.134	0.312
Parameter No. 1	798.15	800.87	1001.55	998.93	37.891	−0.037	0.097
Parameter No. 2	998.56	1000.71	1001.57	998.91	47.305	−0.047	0.121
Parameter No. 3	908.88	887.68	1102.83	1095.45	43.123	−0.043	0.108
Parameter No. 4	858.11	838.31	1102.88	1095.62	40.72	−0.041	0.102

**Table 2:** The measurement results of LTS sensor.

Paramters	Parameter Specification	Parameter No. 1	Parameter No. 2	Parameter No. 3	Parameter No. 4
	50.23	50.14	50.14	50.13	50.14
	49.66	49.87	49.86	49.87	49.87
Measurement	50.34	49.94	49.94	49.94	49.94
results	49.79	50.11	50.11	50.11	50.12
	50.25	50.17	50.16	50.17	50.17
	50.77	50.01	50.03	50.03	50.02
Average	50.17	50.04	50.04	50.04	50.04
